# Consistency of maternal cognitions and principles across the first five months following preterm and term deliveries

**DOI:** 10.1016/j.infbeh.2014.09.005

**Published:** 2014-11

**Authors:** A. Winstanley, R.G. Sperotto, D.L. Putnick, S. Cherian, M.H. Bornstein, M. Gattis

**Affiliations:** a Department of Psychology, University of Cambridge, Cambridge, UK; b School of Psychology, Cardiff University, Cardiff, UK; c *Eunice Kennedy Shriver* National Institute of Child Health and Human Development, National Institutes of Health, Bethesda, MD, USA; d Department of Child Health, University Hospital of Wales, Cardiff, UK

**Keywords:** Parenting, Prematurity, Principles, Cognitions, Caregiving, Infancy

## Abstract

The aims of this study were to examine and compare the development of parenting cognitions and principles in mothers following preterm and term deliveries. Parenting cognitions about child development, including thinking that is restricted to single causes and single outcomes (categorical thinking) and thinking that takes into account multiple perspectives (perspectivist thinking), have been shown to relate to child outcomes. Parenting principles about using routines (structure) or infant cues (attunement) to guide daily caregiving have been shown to relate to caregiving practices. We investigated the continuity and stability of parenting cognitions and principles in the days following birth to 5 months postpartum for mothers of infants born term and preterm. All parenting cognitions were stable across time. Categorical thinking increased at a group level across time in mothers of preterm, but not term, infants. Perspectivist thinking increased at a group level for first-time mothers (regardless of birth status) and tended to be lower in mothers of preterm infants. Structure at birth did not predict later structure (and so was unstable) in mothers of preterm, but not term, infants and neither group changed in mean level across time. Attunement was consistent across time in both groups of mothers. These results indicate that prematurity has multiple, diverse effects on parenting beliefs, which may in turn influence maternal behavior and child outcomes.

## Highlights

• Consistency in group level (continuity) and individual level (stability) was examined longitudinally for caregiving principles (structure and attunement) and cognitions (categorical thinking, perspectivist thinking, complexity of thought) in mothers of preterm and term infants from birth to 5 months old.
• Attunement was continuous and stable in mothers of preterm and term infants.
• Structure was continuous in both groups but stable only in mothers of term infants.
• Complexity of thought was continuous in both groups, perspectivist thinking increased in both groups but only for first-time mothers, and categorical thinking increased only in mothers of preterm infants.
• Categorical thinking, perspectivist thinking, and complexity of thought were stable in mothers of both preterm and term infants.

## 1 Consistency of maternal cognitions and principles across the first five months following preterm and term deliveries

A large literature documents the importance of parenting beliefs about infants and caregiving (Bornstein, 2015, Bornstein et al., 2007, Dichtelmiller et al., 1992, Goodnow, 1988, Miller, 1988, Miller-Loncar et al., 2000, Moorman and Pomerantz, 2008, Pomerantz and Dong, 2006). However, few studies have examined the parenting beliefs of parents of preterm infants, despite preterm deliveries occurring in around 12–13% of live births in the United States and around 5–9% in Europe and other developed countries (Goldenberg, Culhane, Iams, & Romero, 2008). This gap in the literature is significant because infants born prematurely may be at risk due to early non-optimal caregiving environments (e.g., Clark et al., 2008, Feldman and Eidelman, 2006, Forcada-Guex et al., 2006). The current study compared two types of maternal beliefs–cognitions about child development and principles of caregiving–in comparable mothers of term and preterm infants and did so across two time points during infancy. Therefore, this study was able to examine both the nature, as well as the effect, of prematurity on the developmental trajectories of parenting cognitions and principles.

### 1.1 Continuity and stability

One aim of developmental research is to understand how constructs develop across time (Bates & Novosad, 2006; Wohlwill, 1970). In this study, we focused on two approaches to measure the development of parenting cognitions and principles: continuity and stability (Bornstein, 2002). Continuity is defined as consistency in group mean level performance across time. A continuous construct is one in which group means do not differ from one time point to a later time point, whereas changes in mean group performance across time would demonstrate that a construct is discontinuous. Individual differences have a complementary focus on variation around the mean. Stability in individual variation is defined as consistency in the relative rank or standing of individuals within a group across time. A stable construct is one on which some individuals rank at relatively high levels at one point in time and again display at relatively high levels at a later point in time, whereas other individuals display lower levels at both times. An unstable construct is one in which individuals do not maintain their rank order across time.

Studying the continuity and stability of variables provides both a descriptive and explanatory account of development. Continuity and stability not only tell us about individual differences but also about the developmental origins, nature, and future of constructs (Bornstein & Putnick, 2012). Stability and continuity are mainstay concepts in developmental science and represent statistically and theoretically independent spheres of development (Bornstein & Bornstein, 2008). Developmental scientists are not only interested in how constructs manifest themselves but also in group and individual development across time and, therefore, in continuity and stability.

### 1.2 Parenting cognitions and principles

We chose to study the continuity and stability of both maternal cognitions and principles because parenting is multidimensional, modular, and specific (Bornstein, 2002, Bornstein, 2006). That is, different parental behaviors and beliefs serve different functions, have different developmental trajectories, and have different effects on children, with different domains not necessarily related. We selected the cognitions and principles described below based on their documented relations with parenting behavior and practices as well as child outcomes that may be particularly important for preterm infants (see Section 1.3).

First, we studied the complexity of mothers’ thinking about development. Specifically, we focused on two levels of reasoning – *categorical* and *perspectivist thinking* – as well as an additional summary variable that reflects the balance between these two levels – *complexity of thought* (Sameroff & Feil, 1985). Perspectivist thinking reflects flexible reasoning that involves multiple perspectives and takes into account reciprocal influences and transactional perspectives on development, and is therefore more complex. Categorical thinking reflects reasoning that attributes behavior to a single cause and views the child as an extension of parents without individual needs and is therefore less complex. These two levels of complexity of thought describe the broader context of how mothers conceive of children and the parenting role (Miller-Loncar et al., 2000).

A parent's ability to think complexly has been shown to relate to both parental behavior and child outcomes. Parents who think more complexly about child development show more sensitive and responsive parenting behaviors ([Bibr bib0185][Bibr bib0215], Pratt et al., 1993). The preterm and term infants of these parents, in turn, show higher levels of social responsiveness during childhood (Miller-Loncar et al., 2000). However, parents who rely on lower levels of thinking, who have fewer conceptual resources and perspectives to draw on, tend to show a more rigid and authoritarian behavioral style (Deković & Gerris, 1992).

Second, we measured parenting principles of caregiving, which are specific personal codes that guide caregiving during infancy (Winstanley & Gattis, 2013) rather than the general level parents can think about development (as described above). Caregiving principles reflect how parents make decisions about infant care. Specifically, we focused on two principles: *structure* and *attunement*. Structure reflects mothers’ support of schedules and routines to guide their infants’ day-to-day lives. Attunement reflects mothers’ attention to and reliance on their infants’ cues to guide daily caregiving. These two caregiving principles are independent, and therefore some parents support attunement and oppose structure (or vice versa), whereas others support or oppose both principles. Structure and attunement are related to parenting practices. For example, attunement is positively related to bed-sharing, breastfeeding, and holding in parents of infants under 18 months (Winstanley & Gattis, 2013).

### 1.3 Prematurity

There are several reasons to hypothesize that complexity of thought and caregiving principles could be important to understanding the social environment of infants following prematurity. First, the behaviors that have been documented in interactions between mothers and their preterm infants are the same as those that are related to lower perspectivist and higher categorical thinking. That is, mothers of preterm infants have been described as more intrusive and controlling and less sensitive and responsive (Feldman and Eidelman, 2006, Feldman and Eidelman, 2007; Forcada-Guex et al., 2006). The increased intrusiveness and reduced sensitivity seen in mother-preterm infant interactions could therefore reflect differences in maternal cognitions about development (such as, higher levels of categorical thinking). One study did show that mothers of 4-year-olds born preterm tended to score higher on categorical thinking (Pearl & Donahue, 1995). However, we do not yet know how early these differences appear and how categorical and perspectivist thinking develop.

An additional reason to study complexity of thought and caregiving principles following prematurity is that parenting beliefs could be particularly meaningful for preterm infants’ development. For example, using preterm infants’ cues and states (sleepiness, arousal, hunger) to guide caregiving is crucial to ensure that such care is developmentally appropriate, as evidenced by the focus of many NICU-based interventions on new parents learning to use infants’ cues about hunger, distress, and sleepiness (Browne and Talmi, 2005, Graven and Browne, 2008, Kaaresen et al., 2006, Landry et al., 2008). NICU-based interventions have been developed on findings that parents who attend to the behavioral cues of their preterm infants to provide supportive early interactions have infants, and later children, with more positive outcomes (Bozzette, 2007, Landry et al., 1997). Support of the use of infants’ cues in this way is a central focus in the caregiving principle of attunement. In addition, the parenting practices of breastfeeding and holding (in particular, skin-to-skin touch) are advocated when caring for preterm infants (e.g., Flacking, Ewald, & Wallin, 2011; Tessier et al., 1998), and breastfeeding and holding practices are related to stronger support of attunement (Winstanley & Gattis, 2013). Therefore, parents’ support of attunement (with or without structure) could be important for positive infant outcomes following prematurity.

Parenting cognitions and principles have generally been found to be stable in samples of parents of term infants of middle-SES European American background (Cote and Bornstein, 2003, Holden and Miller, 1999, Rubin and Mills, 1992). However, less is known about the stability and continuity of maternal complexity of thought and caregiving principles following preterm deliveries. Measuring complexity of thought and caregiving principles at one time point provides a static picture of how mothers approach and think about caregiving and their child. Looking at just one time point is inadequate because complexity of thought and caregiving principles are likely to change with time as early caregiving moves from the hospital to the home, and as children develop. Very early caregiving of preterm infants often occurs in the hospital, and during this time some parents report feelings that the medical staff are more capable of caring for their preterm infant than they are (Cleveland, 2008, Goldberg and DiVitto, 1983, Howson et al., 2012). In addition, because preterm birth is often unexpected, parents are suddenly forced, ill-prepared, into parenthood, and so they may not have had ample opportunity to attend antenatal classes, read books about parenting and child development, or develop principles about how to care for their infants (Goldberg & DiVitto, 2002). It is therefore important to examine how complexity of thought and caregiving principles change or remain consistent from the days following a premature delivery into later infancy once caregiving routines have been established. As such, prematurity offers the interesting opportunity to understand how parenting complexity of thought and caregiving principles develop under different circumstances.

### 1.4 Methodological issues

This study aimed to chart maternal beliefs following preterm birth, and so child chronological age (calculated from date of birth) was used to schedule visits. This decision was based on our plan to equate amounts of extrauterine experience across dyads at both time points. By contrast, using corrected age (calculated from the due date) to ensure equivalent biological maturity, mothers of preterm and term infants would necessarily differ in the quantity of postnatal and dyadic experience. Corrected age is problematic when studying social development and, in particular, the effects of preterm birth on early parent-infant interactions (Brachfeld et al., 1980, Wilcox et al., 2011).

When studying parenting following premature delivery, it is also imperative to distinguish between prematurity itself and the other factors associated with prematurity (Anderson & Doyle, 2008). For example, preterm birth occurs more often among mothers of low socioeconomic status, who are under 15 years of age, or who have had many pregnancies close together in time (Behrman & Butler, 2006). These demographic factors, without the consideration of premature deliveries, may be related to maternal cognitions and principles. Therefore, differences in beliefs between mothers of preterm and term infants may be ascribable to reasons other than the birth status of their infant. For example, modest relations between complexity of thought and maternal education have been reported (Gutierrez & Sameroff, 1990; Pratt et al., 1993), and lower educational attainment is a risk factor for premature delivery (Behrman & Butler, 2006). In addition, biological risk and neonatal experience of infants (for example, 5-minute Apgar score or days on ventilation) can affect outcomes for children and their social interactions (Aylward, 2002, Hintz et al., 2005, Landry et al., 1997, Vohr et al., 2000). Confounding variables must therefore be carefully explored (Aylward, 2002). To isolate the impact of prematurity on maternal complexity of thought and caregiving principles, we examined multiple demographic and medical covariates for the developmental trajectories of complexity of thought and caregiving principles following preterm and term deliveries.

### 1.5 This study

This study examined how maternal complexity of thought and caregiving principles develop from the days following a premature or term delivery to a time when the parenting role is more established, 5 months later. Therefore, we assessed the continuity and stability of complexity of thought and caregiving principles in mothers of preterm vs. term infants. Our first aim was to examine whether the continuity and stability of maternal complexity of thought and caregiving principles differed by birth status when measured at birth and again 5 months later. We chose to schedule the follow-up data collection at 5 months to ensure parents had become established in the parenting role and infants were settled (St James-Roberts et al., 2006). We expected to see differences in the developmental trajectories of preterm and term mothers’ complexity of thought and caregiving principles from birth to 5 months. Such results would demonstrate that the dynamics of complexity of thought and caregiving principles look different for mothers of preterm and term infants. Our second aim was to examine potential predictors of any observed change in complexity of thought and caregiving principles. To do this, we determined whether medical and demographic factors accounted for any changes observed.

Few studies have systematically examined basic developmental properties of maternal complexity of thought and caregiving principles following premature deliveries. These assessments will increase our understanding of the development of maternal complexity of thought and caregiving principles in general and specifically in response to premature deliveries. Furthermore, this study examined whether prematurity had uniform or differentiated effects on complexity of thought and caregiving principles.

## 2 Method

### 2.1 Participants

A total sample of 105 mothers completed questionnaires within the first month from delivery and 5 months later as part of a study about mothers’ and their preterm (*n* = 41) or term (*n* = 64) infants’ development. Parents of infants born between 30 and 42 weeks gestational age were recruited. Mothers were divided into two groups by the birth status of their infant based on infant's gestational age; infants below 37 completed weeks of gestation were in the preterm sample (up to and including 36 weeks and 6 days; Howson et al., 2012) and those 37 weeks and above were in the term sample. The majority of participants were recruited during the hospitalization period following delivery through the Department of Child Health at University Hospital Wales (UHW, *n* = 90), with the remaining 15 parents recruited through the Cardiff city registry office and other community links, such as the National Childbirth Trust (recruited either soon after delivery or prenatally but completed the questionnaires soon after delivery). An additional 3 dyads were excluded due to a history of maternal depression or placement on the child-in-need register by local authorities to monitor the child due to concerns about the social environment of the child—for example, exposure to domestic abuse. Families were not approached if their infants had serious medical conditions beyond prematurity alone or congenital abnormalities that could affect growth and development, including requiring surgical intervention during hospitalization. Additionally, multiple births and parents under 16 years old were excluded. Suitable infants were identified as fitting inclusion and exclusion criteria through discussions with medical staff (midwife, nurse, or doctor) responsible for their care.

At birth, 148 mothers completed questionnaires. Attrition between birth and 5 months was 29% resulting in the final sample of 105 dyads at 5 months. The majority of parents who did not participate were not contactable at 5 months (53%); others had difficult life circumstances (27%), their infant had developed health problems (10%), or no longer wished to participate (10%).

Table 1 compares health and demographic information about participating mothers and their preterm or term infants. The preterm and term samples did not differ on any of the demographic variables (infant birth order, maternal age, ethnicity, marital status, maternal education, and family income) or on 5-min Apgar scores. The samples naturally differed on medical status—preterm infants were born at younger gestational age and lower birthweight and spent more days on ventilation and in the hospital after birth. These variables were all checked as potential covariates and predictors of change in complexity of thought and caregiving principles.

**Table 1:** Demographic and medical characteristics of the sample.

			Preterm	Term	Difference
Demographic characteristics					
Infant chronological age (days)	Newborn	*M* (*SD*)	10.56 (9.63)	10.14 (9.98)	*t*(103) = 0.21, *p* = 0.831, *d* = 0.05
	5 months	*M* (*SD*)	152.32 (5.91)	153.22 (6.72)	*t*(103) = −0.70, *p* = 0.484, *d* = −0.14
Infant gender	Female	*N* (%)	16 (39)	29 (45)	*χ*^2^(1, *N* = 105) = 0.40, *p* = 0.525
	Male	*N* (%)	25 (61)	35 (55)	
Birth order	First born	*N* (%)	23 (56)	42 (66)	*χ*^2^(1, *N* = 105) = 0.96, *p* = 0.327
	Later born	*N* (%)	18 (44)	22 (34)	
Number of siblings		*M* (*SD*)	0.59 (0.97)	0.44 (0.73)	*t*(103) = 0.89, *p* = 0.378, *d* = 0.17
Maternal age (years)		*M* (*SD*)	31.63 (5.15)	32.19 (4.41)	*t*(103) = −0.59, *p* = 0.558, *d* = −0.12
Marital status	Single	*N* (%)	8 (20)	9 (14)	*χ*^2^(2, *N* = 105) = 0.71, *p* = 0.700
	Co-habiting	*N* (%)	6 (15)	12 (19)	
	Married	*N* (%)	27 (66)	43 (67)	
Maternal education	GCSEs	*N* (%)	5 (12)	6 (9)	*χ*^2^(3, *N* = 104) = 1.88, *p* = 0.598
	A-Levels	*N* (%)	8 (19)	7 (11)	
	Bachelor's	*N* (%)	13 (32)	25 (40)	
	Postgraduate	*N* (%)	15 (37)	25 (40)	
Household income	Less than £14,999	*N* (%)	8 (20)	4 (6)	*χ*^2^(2, *N* = 102) = 4.73, *p* = 0.094
	£15,000–£39,999	*N* (%)	11 (27)	16 (26)	
	Over £40,000	*N* (%)	21 (53)	42 (68)	
Maternal ethnicity	Caucasian	*N* (%)	35 (95)	61 (97)	*χ*^2^(1, *N* = 100) = 0.30, *p* = 0.625
	Other	*N* (%)	2 (5)	2 (3)	

Infant medical characteristics					
Gestational age		*M* (*SD*)	34.45 (1.70)	39.91 (1.43)	*t*(103) = −17.69, *p* < 0.001, *d* = −3.48
Birthweight (g)		*M* (*SD*)	2167.66 (467.85)	3436.85 (568.48)	*t*(100) = −11.78, *p* < 0.001, *d* = −2.44
Age at discharge (days)		*M* (*SD*)	14.26 (12.49)	2.76 (3.01)	*t*(40.94) = 5.64, *p* < 0.001, *d* = 1.27
5-min Apgar		*M* (*SD*)	9.26 (0.88)	9.36 (1.47)	*t*(92) = −0.41, *p* = 0.685, *d* = −0.08
Ventilation (number of days)		*M* (*SD*)	1.54 (3.02)	0.08 (0.45)	*t*(35.12) = 2.83, *p* = 0.008, *d* = 0.96

*Note. N*s for the preterm and term sample were 41 and 64, respectively. Data were missing for maternal education for 1 term infant, and for family income for 1 preterm and 2 term infants, for maternal ethnicity for 4 preterm infants and 1 term infant, for birthweight for 1 preterm infant and 2 term infants, for age at discharge for 2 preterm infants and 5 term infants, for 5-min Apgar scores for 2 preterm and 9 term infants, and for days of ventilation for 6 preterm and 16 term infants.

### 2.2 Procedures

All study procedures were reviewed by the Cardiff University School of Psychology's research ethics committee, the National Health Service's Research & Development, and Local Research Ethics Committee. Mothers consented to participate soon after delivering their baby at which time they completed the Baby Care Questionnaire (BCQ; Winstanley & Gattis, 2013) and Concepts of Development Questionnaire (CODQ; Sameroff & Feil, 1985) for the first time. Mothers completed these two questionnaires again 5 months later. Health and demographic information was collected from a combination of a self-report measure completed by the mother and inspection of medical records by research assistants. Five-month study visits were scheduled based on postnatal chronological age with a window of ±15 days. Gifts were given to infants and parents for participation (worth approximately USD $10).

### 2.3 Principal measures

#### 2.3.1 The Baby Care Questionnaire (BCQ)

The BCQ (Winstanley & Gattis, 2013) asks parents to rate 30 statements about caregiving on a 4-point Likert scale ranging from *strongly disagree* (1) to *strongly agree* (4). The BCQ contains 2 subscales—structure and attunement. Subscale scores were calculated by averaging across relevant items (17 items for structure and 13 items for attunement). Structure represents parent support of regularity and routines in their infant's daily life. For example, *It is important to introduce a sleeping schedule as early as possible*. Structure showed adequate internal consistency at birth (preterm: *α* = 0.81; term: *α* = 0.87) and 5 months (preterm: *α* = 0.79; term: *α* = 0.84). Attunement represents parent trust and attention to their infant's cues and support of close physical contact. For example, *Responding quickly to a crying baby leads to less crying in the long run*. Three items for the attunement subscale had poor distributions and did not relate with overall attunement or other items making up the attunement subscale and so were not used in calculating the average attunement score. The resulting 10-item attunement scale showed adequate internal consistency at birth (preterm: *α* = 0.60; term: *α* = 0.75) and 5 months (preterm: *α* = 0.68; term: *α* = 0.75).

#### 2.3.2 Concepts of Development Questionnaire (CODQ)

The CODQ (Sameroff & Feil, 1985) asks parents to rate 20 statements about child development on a 4-point Likert scale ranging from *strongly disagree* (0) to *strongly agree* (3). The CODQ measures parent cognitions about child development, in particular parent ability to think complexly about children. The CODQ contains two subscales – categorical and perspectivist – and a summary scale of complexity. For each subscale an average score is calculated. At the categorical level, parent cognitions are restricted to single determinants and single outcomes. For example, *Parents must keep to their standards and rules no matter what their child is like*. Three items for the categorical subscale had poor distributions and did not relate with the subscale overall or with the other items making up the subscale and so were not used to calculate the categorical subscale score. The remaining 7-item categorical subscale showed adequate internal consistency at birth (preterm: *α* = 0.68; term: *α* = 0.77) and 5 months (preterm: *α* = 0.65; term: *α* = 0.53). At the perspectivist level, child development is viewed from multiple perspectives, allowing parents to understand that multiple factors can interact and change over time to result in different outcomes. For example, *Parents change in response to their children*. Cognizing at the perspectivist level allows parents to view and evaluate a large range of developmental possibilities. Three items for the perspectivist subscale had poor distributions and did not relate with the subscale overall or with the other items making up the subscale, so were not used to calculate the perspectivist subscale score. The remaining 7-item perspectivist subscale showed adequate internal consistency at birth (preterm: *α* = 0.60; term: *α* = 0.65) and 5 months (preterm: *α* = 0.60; term: *α* = 0.67). Previous studies have found similar internal consistency coefficients when using the CODQ (e.g., [Bibr bib0025][Bibr bib0185], Lee, 2005, Manlove et al., 2008).

Complexity is calculated as (perspectivist − categorical + 3.0)/2. Therefore, complexity represents the balance between categorical and perspectivist thinking and also ranges from 0 to 3. To ensure the BCQ and CODQ used equivalent scales, after calculating complexity, all subscales of the CODQ were transformed to range from 1 to 4 (by adding 1 to all scores). Therefore, a complexity score of 4 means that parents strongly agree with items related to the perspectivist subscale and strongly disagree with items related to the categorical subscale. Conversely, a complexity score of 1 means that parents strongly disagree with items related to the perspectivist subscale and strongly agree with items related to the categorical subscale.

Correlations among maternal structure, attunement, categorical, perspectivist, and complexity for birth and 5-month variables by birth status indicated that structure was not related to any of the cognitions, but attunement was positively related to perspectivist scores and to a smaller extent complexity scores. However, the correlation coefficients did not indicate singularity for any of these measures (shared variance = 0–28%) and so were treated independently.

### 2.4 Descriptive and explanatory variables

Mothers completed a demographic questionnaire after delivery that collected information about demographic variables, previous pregnancies, and the current or most recent pregnancy and delivery. In addition, researchers accessed the medical records of the infant after discharge and created records of the infants’ health during their hospitalization.

## 3 Results

### 3.1 Analysis plan

Prior to data analysis, distributions of categorical thinking, perspectivist thinking, complexity of thought, structure, and attunement at both time points were examined for normality, homogeneity of variance, and influential outliers. All variables met assumptions for parametric tests.

The first aim was to assess the continuity and stability of complexity of thought and caregiving principles in mothers of preterm and term infants. Therefore, the effects of child age (birth vs. 5 months) and birth status (preterm vs. term) were tested using repeated-measures Analysis of Variance (RM-ANOVA). Child age (birth vs. 5 months) was treated as a within-subjects variable and birth status (preterm vs. term) was treated as a between-subjects variable. In addition, stability estimates were reported across ages for maternal complexity of thought and caregiving principles by birth status using correlation. Table 2 presents means and standard deviations for maternal complexity of thought and caregiving principles by infant age and birth status. Table 2 also presents correlations between subscales at birth and 5 months by infant birth status. Follow-up analyses were run to control for mothers’ previous experience of having a child (birth order: firstborn vs. laterborn) and having previously had a preterm delivery (no vs. yes). The uncontrolled analyses are reported below as all results controlling for experience of mothers with previous children were the same as uncontrolled analyses except where noted.

**Table 2:** Descriptive statistics for parenting cognitions (CODQ) and principles (BCQ) by infant birth status.

	Preterm infants			Term infants		
	Birth	5 months	*r*	Birth	5 months	*r*
	*M* (*SD*)	*M* (*SD*)		*M* (*SD*)	*M* (*SD*)	
CODQ						
Categorical	1.63 (0.34)	1.82 (0.29)	0.52***	1.69 (0.33)	1.74 (0.29)	0.35**
Perspectivist	2.77 (0.34)	2.91 (0.32)	0.50***	2.91 (0.35)	3.00 (0.34)	0.55***
Complexity	3.07 (0.25)	3.05 (0.22)	0.50***	3.11 (0.27)	3.13 (0.24)	0.50***

BCQ						
Structure	2.76 (0.27)	2.76 (0.29)	0.27	2.64 (0.35)	2.66 (0.38)	0.66***
Attunement	2.87 (0.26)	2.90 (0.30)	0.57***	2.96 (0.34)	2.98 (0.35)	0.63***

*Note. N*s for the preterm and term sample were 41 and 64, respectively.

** *p* < 0.005.

*** *p* < 0.001.

The next aim was to identify what variables may account for any developmental changes observed in complexity of thought and caregiving principles. Multiple regression analyses were used to examine predictors of differential stability across groups, and RM-ANCOVAs were used to examine predictors of discontinuity. The predictors of change in mean level or discontinuity were examined using RM-ANCOVAs. First, the main effects of infant age (birth vs. 5 months) and birth status, and the effect of the infant age × birth status interaction on cognitions or principles at 5 months were examined. Then, covariates were included to examine their effect on the significance of the main effect of age and the infant age × birth status interaction. Reducing either the main effect of age or the infant age × birth status interaction to nonsignificance would indicate that the covariates, at least in part, accounted for the discontinuity of maternal cognitions or principles. For the predictors of differential stability of cognitions or principles, we used a similar process with multiple regressions. In the first step, cognitions or principles at birth (centered), birth status (preterm vs. term), and the interaction between cognitions or principles at birth and birth status were included as predictors, and cognitions or principles at 5 months was the outcome variable. Covariates were then included in the second step to examine whether their inclusion attenuated the cognitions or principles at birth or interaction term to nonsignificance. Potential health and demographic covariates were selected by examining zero-order correlations with 5-month scores for cognitions or principles of interest.

### 3.2 Complexity of thought

For categorical thinking, there was a main effect of age, *F*(1, 103) = 12.28, *p* < 0.001, *η*_*p*_^2^ = 0.107, no main effect of birth status, *F*(1, 103) = 0.00, *p* = 0.960, *η*_*p*_^2^ = 0.002, and a significant interaction between infant age and birth status, *F*(1, 103) = 4.34, *p* = 0.040, *η*_*p*_^2^ = 0.040. Simple effects analyses examined the interaction between infant age and birth status on categorical thinking. A main effect of age was found for mothers of preterm, *F*(1, 40) = 15.36, *p* < 0.001, *η*_*p*_^2^ = 0.277, but not term infants, *F*(1, 63) = 1.17, *p* = 0.283, *η*_*p*_^2^ = 0.018. Categorical thinking was continuous in mothers of term, but not preterm, infants. Mothers of preterm and term infants did not differ in their categorical thinking at birth, *F*(1, 103) = 1.03, *p* = 0.312, *η*_*p*_^2^ = 0.010, or 5 months later, *F*(1, 103) = 1.61, *p* = 0.208, *η*_*p*_^2^ = 0.015. Mothers of preterm infants increased their categorical thinking with age, whereas mothers of term infants did not change with age.

We examined possible explanations for the discontinuity of categorical scores in mothers of preterm infants. Apgar scores at 5 min, number of siblings, and whether the mother was living with a partner (no vs. yes) were included as covariates because these variables were related to categorical thinking at 5 months. Apgar scores at 5 min were not recorded in medical records for 11 infants (2 preterm and 9 term), and therefore these dyads were excluded, resulting in a final sample of 94 (39 preterm and 55 term). Table 3 presents the *F*, *p*, and *η*_*p*_^2^ values for step 1—infant age, birth status, and infant age × birth status interaction – and step 2—adding covariates – in predicting categorical thinking. In step 1, there was a significant main effect of age, *F*(1, 92) = 11.83, *p* < 0.001, *η*_*p*_^2^ = 0.114, but no main effect of birth status, *F*(1, 92) = 0.32, *p* = 0.573, *η*_*p*_^2^ = 0.003, and a trend level interaction between infant age and birth status, *F*(1, 92) = 3.77, *p* = 0.055, *η*_*p*_^2^ = 0.039, on categorical scores. Adding covariates attenuated the main effect of age to nonsignificance, *F*(1, 89) = 0.15, *p* = 0.705, *η*_*p*_^2^ = 0.002 (a 0.112 reduction in the *η*_*p*_^2^), but only very slightly reduced the interaction between age and birth status, *F*(1, 89) = 3.46, *p* = 0.066, *η*_*p*_^2^ = 0.037 (a 0.002 reduction in the *η*_*p*_^2^). None of the covariates independently predicted discontinuity in categorical thinking. The RM-ANCOVAs for mothers of preterm and term infants individually showed that the main effect of age was nonsignificant for both groups (preterm: *F*(1, 35) = 0.97, *p* = 0.332, *η*_*p*_^2^ = 0.027; term: *F*(1, 51) = 0.41, *p* = 0.523, *η*_*p*_^2^ = 0.008). The only difference in the RM-ANCOVAs was that Apgar scores at 5 min independently predicted categorical scores at 5 months for mothers of preterm infants, *F*(1, 35) = 4.43, *p* = 0.042, *η*_*p*_^2^ = 0.112 (higher Apgar scores related to higher categorical thinking), but not term infants, *F*(1, 51) = 0.01, *p* = 0.932, *η*_*p*_^2^ = 0.000. Together, Apgar at 5 min, number of siblings, and mother living with a partner accounted for discontinuity in categorical thinking for both groups, but only Apgar scores at 5 min independently predicted categorical scores at 5 months in mothers of preterm infants.

**Table 3:** Predictors of discontinuity in categorical and perspectivist thinking.

	Step					
	1			2		
	*F*	*p*	*η*_*p*_^2^	*F*	*p*	*η*_*p*_^2^
Categorical thinking at 5 months						
Age (birth vs. 5 months)	11.83	<0.001	0.114	0.15	0.705	0.002
Birth status (preterm vs. term)	0.32	0.573	0.003	0.44	0.510	0.005
Age × birth status	3.77	0.055	0.039	3.46	0.066	0.037
Apgar at 5 min				1.07	0.303	0.012
Number of siblings				1.86	0.177	0.020
Mother living with partner				0.86	0.357	0.010

Perspectivist thinking at 5 months						
Age (birth vs. 5 months)	16.20	<0.001	0.210	2.83	0.098	0.046
Birth status (preterm vs. term)	2.19	0.144	0.035	0.10	0.752	0.002
Age × birth status	0.84	0.364	0.014	0.94	0.338	0.016
Highest maternal qualification				1.57	0.215	0.026
Antenatal class attendance				1.27	0.265	0.021
Breastfeeding at birth				2.85	0.097	0.047

*Note*. Data were missing for 11 dyads for Apgar scores at 5 min so there was a final sample of 39 preterm and 55 term infants for the categorical thinking analyses. Therefore, degrees of freedom for step 1 were (1, 92) and for step 2 were (1, 89). Data were missing for 1 dyad for highest maternal qualification, 1 for antenatal class attendance and 1 for breastfeeding at birth so there was a final sample of 40 preterm and 62 term infants for the perspectivist thinking analyses. Therefore, degrees of freedom for step 1 were (1, 100) and for step 2 were (1, 97).

For perspectivist thinking, there was a main effect of age, *F*(1, 103) = 12.53, *p* < 0.001, *η*_*p*_^2^ = 0.108, a main effect of birth status at a trend level, *F*(1, 103) = 3.70, *p* = 0.057, *η*_*p*_^2^ = 0.035, but no significant interaction between infant age and birth status, *F*(1, 103) = 0.48, *p* = 0.491, *η*_*p*_^2^ = 0.005. Mean differences demonstrated that perspectivist thinking was lower, on average, at birth (mean difference = −0.12, *SE* = 0.03, *p* < 0.001) and in mothers of preterm infants (mean difference = −0.11, *SE* = 0.06, *p* = 0.057). When controlling for previous children and previous preterm birth, the main effect of age remained, *F*(1, 99) = 17.39, *p* < 0.001, *η*_*p*_^2^ = 0.149, as did the main effect of birth status, *F*(1, 99) = 4.00, *p* = 0.048, *η*_*p*_^2^ = 0.039. However, there was also a significant interaction between age and birth order, *F*(1, 99) = 5.11, *p* = 0.026, *η*_*p*_^2^ = 0.049. Simple effects analyses indicated that the main effect of age was only significant for mothers of firstborns, *F*(1, 61) = 20.39, *p* < 0.001, *η*_*p*_^2^ = 0.251, and not mothers of laterborns, *F*(1, 36) = 0.02, *p* = 0.894, *η*_*p*_^2^ = 0.000. Mothers of firstborns increased in their perspectivist thinking from birth to 5 months (birth: *M* = 2.78, *SD* = 0.35; 5 months: *M* = 2.96, *SD* = 0.35) but perspectivist thinking did not change for mothers of laterborns (birth: *M* = 2.92, *SD* = 0.35; 5 months: *M* = 2.94, *SD* = 0.35) for both groups of mothers. In addition, mothers of preterm infants were lower on perspectivist thinking than mothers of term infants.

We examined potential explanations for the discontinuity of perspectivist scores in the mothers of firstborn preterm and term infants. Mother's highest educational qualification, attending antenatal classes (no vs. yes), and breastfeeding at birth (no vs. yes) were included as covariates because these variables were related to perspectivist thinking at 5 months. Only first-time mothers were included, and one dyad was excluded due to missing data for breastfeeding at birth, resulting in a final sample of 63 (22 preterm and 41 term). Table 3 presents the *F*, *p*, and *η*_*p*_^2^ values for step 1—infant age, birth status, and infant age × birth status interaction – and step 2—adding covariates – in predicting perspectivist thinking. In step 1, there were main effects of age, *F*(1, 61) = 16.20, *p* < 0.001, *η*_*p*_^2^ = 0.210, but no significant main effect of birth status, *F*(1, 61) = 2.19, *p* = 0.144, *η*_*p*_^2^ = 0.035, or interaction between infant age and birth status, *F*(1, 61) = 0.84, *p* = 0.364, *η*_*p*_^2^ = 0.014, on perspectivist scores. Adding covariates attenuated the main effects of age, *F*(1, 58) = 2.83, *p* = 0.098, *η*_*p*_^2^ = 0.046 (a 0.164 reduction in the *η*_*p*_^2^) to nonsignificance. None of the covariates independently predicted perspectivist thinking. Therefore, highest maternal qualification, attending antenatal classes, and breastfeeding together accounted for the discontinuity of perspectivist thinking.

Finally, there were no main effects of age, *F*(1, 103) = 0.01, *p* = 0.937, *η*_*p*_^2^ = 0.000, or birth status, *F*(1, 103) = 1.82, *p* = 0.181, *η*_*p*_^2^ = 0.017, or interaction between infant age and birth status, *F*(1, 103) = 0.93, *p* = 0.336, *η*_*p*_^2^ = 0.009, for complexity of thought. Complexity of thought did not differ by infant age or birth status and was therefore continuous for mothers of preterm and term infants.

Stability was found for all three CODQ variables in mothers of term and preterm infants (Table 2). *Z*-tests indicated that the stability coefficients did not differ between mothers of preterm and term infants for categorical scores (*z* = 1.02, *p* = 0.308, two-tailed test), perspectivist scores (*z* = −0.33, *p* = 0.741, two-tailed test), or complexity scores (*z* = 0.00, *p* = 1.00, two-tailed test).

### 3.3 Caregiving principles

For structure, there was no main effect of age, *F*(1, 103) = 0.12, *p* = 0.729, *η*_*p*_^2^ = 0.001, or birth status, *F*(1, 103) = 3.28, *p* = 0.073, *η*_*p*_^2^ = 0.031, or interaction between infant age and birth status, *F*(1, 103) = 0.18, *p* = 0.671, *η*_*p*_^2^ = 0.002. When controlling for previous children and previous preterm birth, there was a significant main effect of birth status, *F*(1, 99) = 3.96, *p* = 0.050, *η*_*p*_^2^ = 0.038, but still no main effect of age, *F*(1, 99) = 0.83, *p* = 0.364, *η*_*p*_^2^ = 0.008, or interaction between infant age and birth status, *F*(1, 99) = 0.07, *p* = 0.789, *η*_*p*_^2^ = 0.001. The main effect of birth status reflected that mothers of preterm infants (*M* = 2.77, *SD* = 0.30) supported structure more, on average, than mothers of term infants (*M* = 2.65, *SD* = 0.30).

For attunement, there was no main effect of age, *F*(1, 103) = 0.89, *p* = 0.349, *η*_*p*_^2^ = 0.009, or birth status, *F*(1, 103) = 2.06, *p* = 0.154, *η*_*p*_^2^ = 0.020, or interaction between infant age and birth status, *F*(1, 103) = 0.04, *p* = 0.838, *η*_*p*_^2^ = 0.000. Therefore, structure and attunement did not differ across age or birth status and were continuous for mothers of preterm and term infants. However, when controlling for previous experience of caregiving, mothers of preterm infants were higher on structure than mothers of term infants.

Stability was found for structure for mothers of term infants but not mothers of preterm infants (Table 2). *Z*-tests indicated that the stability of structure was significantly lower for mothers of preterm infants than term infants (*z* = −2.50, *p* = 0.012, two-tailed test). Stability was also found for attunement for mothers of preterm and term infants (Table 2). The stability of attunement did not differ between mothers of preterm and term infants (*z* = −0.45, *p* = 0.653, two-tailed test).

We examined potential explanations for the differential stability of structure in mothers of preterm and term infants. Family income was included as a covariate because this variable was related to structure at 5 months. Three dyads were missing data on family income and therefore were excluded from these analyses resulting in a final sample of 102 (40 preterm and 62 term). Table 4 presents the regression coefficients for step 1—structure at birth (centered), birth status, and structure at birth × birth status interaction – and step 2—adding family income – in predicting structure at 5 months. Step 1 accounted for 34% of the variance in structure scores at 5 months, *R*^2^ = 0.34, *F*(3, 98) = 16.72, *p* < 0.001. Neither structure at birth nor birth status predicted structure at 5 months, but the interaction between structure and birth status predicted structure at 5 months. This result reflects the earlier finding that structure was only stable in mothers of term infants and not in mothers of preterm infants. Step 2 did not account for significantly more variance than step 1, *ΔR*^2^ = 0.00, *F*(1, 97) = 0.53, *p* = 0.467. Adding family income as a covariate attenuated the interaction term to nonsignificance (model 1: *β* = −0.21, *p* = 0.037; model 2: *β* = −0.19, *p* = 0.065) but only just (a 0.02 absolute reduction in the *β*). Family income did not independently predict instability in structure. Therefore, family income accounts for some of the differential stability in structure between mothers of preterm and term infants but only to a limited degree.

**Table 4:** Predictors of differential instability in structure of mothers of preterm and term infant.

	Step			
	1		2	
	*B* (*SE*)	*β*	*B* (*SE*)	*β*
Structure at 5 months				
Structure at birth (centered)	0.70 (0.10)	0.66**	0.70 (0.11)	0.66**
Birth status (0 = term, 1 = preterm)	0.03 (0.06)	0.05	0.02 (0.06)	0.03
Structure at birth × birth status	−0.43 (0.20)	−0.21*	−0.40 (0.21)	−0.19^a^
Birth order			−0.02 (0.06)	−0.03
Family income			−0.03 (0.04)	−0.06
*R*^2^	0.34		0.34	

*F* for change in *R*^2^	*F*(3, 98) = 16.72, *p* < 0.001		*F*(5, 96) = 10.03, *p* = 0.467	

*Note*. Data were missing for 3 dyads for family income so there was a final sample of 40 preterm and 62 term infants.

* *p* < 0.05.

** *p* < 0.001.

a *p* = 0.065.

## 4 Discussion

The first aim of the study was to examine developmental trajectories of maternal complexity of thought and caregiving principles following preterm and term deliveries in the first 5 months of their infant's life. We found prematurity had a differentiated effect on complexity of thought and caregiving principles, with different levels of complexity of thought and caregiving principles affected differently. The second aim was to examine demographic and medical variables that might explain changes in complexity of thought and caregiving principles. Below we first discuss the findings related to complexity of thought and then caregiving principles.

### 4.1 Complexity of thought

Categorical thinking was continuous for mothers of term, but not preterm, infants. Mothers of preterm infants showed increasing levels of categorical thinking from the delivery of their infant to 5 months later (and therefore an increase in less complex thinking). In combination, Apgar scores at 5 min, number of siblings, and whether mothers were living with a partner accounted for the discontinuity of categorical thinking in mothers of preterm infants. The only independent predictor of categorical thinking was 5-min Apgar scores for mothers of preterm, but not term, infants. For mothers of preterm infants, higher 5-min Apgar scores were related to higher categorical thinking scores at 5 months but not at birth. However, it must be noted that 5-min Apgar scores of the preterm infants reflected the low-risk nature of the sample, as scores ranged from 7 to 10 (but around 86% scored 9 or 10). Therefore, this finding needs to be further examined in a higher risk sample with a wider range of Apgar scores. For both groups of mothers of firstborn infants, perspectivist thinking increased over time and overall levels were lower in all mothers of preterm infants regardless of parity. The discontinuity of perspectivist thinking for first-time mothers was accounted for by a combination of highest maternal qualification, having attended antenatal classes, and breastfeeding at birth (however, none of these variables independently predicted perspectivist thinking). Finally, complexity of thought was continuous for both birth status groups. Stability was found at equal levels for all three complexity of thought variables across birth status groups.

Despite equal levels of complexity of thought, prematurity had an impact on categorical and perspectivist thinking. Categorical thinking increased in mothers of preterm infants from birth to 5 months but remained continuous in mothers of term infants. In addition, mothers of preterm infants were less able (at trend levels) to demonstrate perspectivist thinking. However, complexity of thought did not differ by the birth status or age of the infant. Despite being able to think as complexly about child development as mothers of term infants, mothers of preterm infants appear to increasingly rely on lower levels of thinking. This result could have important implications because lower levels of thinking have been shown to relate to more rigid and authoritarian parenting (Deković & Gerris, 1992), and more complex levels of thinking have been shown to relate to warm and sensitive parenting (Landry et al., 1996, Miller-Loncar et al., 2000, Pratt et al., 1993). Furthermore, this result could inform the design of NICU interventions. That is, early interventions could focus on helping mothers to take multiple, flexible perspectives when thinking about children and development (and so become less reliant on lower levels of thinking). Further work needs to examine whether the similar levels and trajectories of complexity of thought for mothers of preterm infants can prove protective despite differences in categorical and perspectivist thinking (compared to mothers of term infants). Therefore, relations between complexity of thought (both overall level and trajectories) with later parenting behaviors and child outcomes need to be studied.

### 4.2 Caregiving principles

Structure and attunement were both stable and continuous in mothers of term infants. In contrast, structure was continuous but unstable, and attunement was continuous and stable, for mothers of preterm infants. Therefore, only the caregiving principle of structure appeared to be affected by premature birth. In mothers of preterm infants, structure at birth did not predict structure 5 months later. Therefore, mothers of preterm infants appeared to change their support of structure over the first 5 months but not in a uniform way (i.e., no mean increase or decrease). The differential stability of structure by birth status was reduced to nonsignificance by controlling for family income. However, the reduction of the interaction term of structure at birth × birth status was small, and family income did not add to the variance accounted for in the regression model. Therefore, this result should be considered with caution. Perhaps characteristics of the infant (for example, sleep state stability or clarity of cues) would provide a better explanation of the differential stability of structure than demographic or medical factors. For example, it could be that the lower stability of structure following preterm delivery reflects that these mothers are more willing to be flexible in structure and base that principle on their infant's willingness or ability to fit into a schedule. Alternatively, as preterm birth is often unexpected and accompanied by periods of hospitalization, mothers’ support of structure at birth may not be a representation of their true principles but instead reflect the structure of the hospital and medical staff. As such, mothers of preterm infants may only develop their own principles about structure on leaving the hospital and becoming fully responsible for their infants’ daily care. Both of these hypotheses fit with findings that, when controlling for previous experience with children, structure appeared to be higher in mothers of preterm, as compared to term, infants. Future work should examine such hypotheses.

Attunement, in comparison, did not appear to be affected by premature deliveries as attunement showed stability and continuity for mothers of preterm and term infants. Many interventions implemented following premature deliveries or during NICU stays focus on teaching parents to respond to the cues and unique characteristics of their infant ([Bibr bib0085][Bibr bib0180], Landry et al., 2008). In addition, more optimal outcomes have been found for children who had parents who followed their interests and cues as infants (Landry et al., 1997). Understanding a parent's support of attunement at the start of one of these interventions or following a premature delivery may prove useful in planning how to best approach helping parents. For example, parents who already support the principle of attunement may only require guidance in identifying their infants’ unique cues, whereas parents who show little or no support of attunement may first need the significance of using their infants’ cues to guide caregiving explained. Therefore, this finding of similar levels and trajectories of attunement demonstrates that mothers of preterm infants, at least in principle, do not differ from mothers of term infants in the value they place in using and trusting infant cues and signals to guide caregiving. Therefore, attunement may reflect an enduring caregiving principle of parents that is less affected by external forces, whereas structure is more responsive to environmental factors. More work is needed to examine the predictors of these principles.

### 4.3 Limitations

There were relatively high levels of attrition between data collection at delivery and at 5 months. Loss of participants was primarily due to difficulties contacting families when attempting to schedule the 5-month visit. In addition, this sample relied on families consenting to take part in the study and as such was not representative of all preterm infants born in UHW. The subscales of the CODQ showed relatively low internal consistency; however, these consistency estimates are similar to those reported previously with samples of parents of term, as well as preterm, infants (e.g., [Bibr bib0025][Bibr bib0185], Lee, 2005, Manlove et al., 2008).

Future work might include a larger, more heterogeneous sample. The current sample was relatively homogeneous, including primarily white mothers of higher income and education. Furthermore, we excluded three families due to a history of depression or concerns about the social environment of the child. We were unable to include these families in the sample because the small number meant that we were not able to control for these social risk factors. Future studies, with larger, more diverse samples, should include such variables in analyses. This study is, however, an important first step to examining the effects of infant prematurity on the development of maternal cognitions and principles.

### 4.4 Conclusions

This study demonstrates differences in the trajectories of some of the studied cognitions and caregiving principles following preterm, as compared to term, deliveries. The studied maternal cognitions and caregiving principles were not affected equally by prematurity. Mothers increasingly relied on categorical thinking in establishing the caregiving role for their preterm infants and were less stable in reports of structuring their caregiving. However, both structure and categorical thinking showed similar levels for mothers of preterm and term infants at both time points. This study highlights the need to examine developmental trajectories of cognitions and principles. In addition to differences in the trajectories of complexity of thought and caregiving principles, mothers of preterm infants tended to report lower perspectivist thinking than mothers of term infants following delivery and after 5 months of caring for their infant.

These results provide insight into the development of complexity of thought and caregiving principles following premature deliveries and tell us about the broader context in which cognitions about child development and caregiving principles develop in parents. For example, first-time mothers (at a group level) increased in their perspectivist thinking as their infants grew older, while mothers of laterborns showed consistency across time. In addition, given that different cognitions about child development and caregiving principles showed different trajectories these results further highlight that parenting is multidimensional (Bornstein and Cote, 2004, Bornstein and Tamis-LeMonda, 1990, Bornstein et al., 2008). Further work needs to examine the change and consistency of these cognitions about child development and caregiving principles later in infancy, as well as their impact on parenting behaviors and child outcomes.

## Author note

Alice Winstanley, Department of Psychology, University of Cambridge; Rebecca G. Sperotto and Merideth Gattis, School of Psychology, Cardiff University; Diane L. Putnick and Marc H. Bornstein, *Eunice Kennedy Shriver* National Institute of Child Health and Human Development; and Shobha Cherian, Department of Child Health, University Hospital of Wales.
